# Comparative Evaluation of Complete Mesocolic Excision Versus Conventional Right Hemicolectomy in Right-Sided Colon Cancer: A Retrospective Observational Study

**DOI:** 10.7759/cureus.84369

**Published:** 2025-05-18

**Authors:** Dhanalakshmi Kadirvel, Srinath Ganesan, Ilango Parthasarathy, Gowtham Karthik V, Mahesh K G

**Affiliations:** 1 General Surgery, Sree Balaji Medical College & Hospital, Chennai, IND; 2 Surgical Oncology, Sree Balaji Medical College & Hospital, Chennai, IND; 3 Surgical Oncology, Sri Ramachandra Institute of Higher Education and Research, Chennai, IND

**Keywords:** central vascular ligation, complete mesocolic excision, conventional right hemicolectomy, intracorporeal anastomosis, right-sided colon cancer, surgical oncology

## Abstract

Background

Complete mesocolic excision (CME) with central vascular ligation (CVL) has emerged as an advanced surgical technique aiming to improve oncological outcomes in right-sided colon cancer. This study aims to compare the surgical and short-term oncological outcomes between CME and conventional right hemicolectomy (CON) performed in a tertiary care center.

Methods

A retrospective observational study was conducted between January 2021 and December 2024 in Sree Balaji Medical College & Hospital, a high-volume tertiary care center in Chennai, India, including 180 patients with right-sided colon cancer. Patients were categorized into two groups based on the surgical technique: CME (n = 94) and CON (n = 86). Parameters assessed included lymph node harvest, operative time, postoperative complications, type of anastomosis (intracorporeal vs. extracorporeal), and stapling technique (Barcelona vs. standard). Statistical analysis was performed using IBM SPSS Statistics, with a significance threshold set at p < 0.05.

Results

The CME group exhibited a significantly higher lymph node yield (≥12 nodes in 94.7% vs. 79.1%, p < 0.001) and increased adoption of intracorporeal anastomosis (44.7% vs. 14.0%, p = 0.001) and Barcelona stapling technique (35.1% vs. 4.7%, p < 0.001). Operative time was longer for CME (156 ± 47 minutes vs. 112 ± 35 minutes), although postoperative morbidity rates were comparable between groups.

Conclusion

CME with CVL offers superior lymph node harvest and facilitates minimally invasive anastomotic techniques without increasing postoperative complications. It should be considered the standard of care in experienced centers.

## Introduction

Complete mesocolic excision (CME) has emerged as a refined surgical technique for the management of right-sided colon cancer, drawing conceptual parallels from total mesorectal excision (TME) widely adopted for rectal cancer. First described by Hohenberger et al. in 2009 [1], CME involves sharp dissection along the embryological planes to mobilize the mesocolon intact, combined with central vascular ligation (CVL) of the ileocolic and right colic vessels at their origin. The goal of CME is to achieve oncologically superior resections with intact mesocolic envelopes, better lymphovascular clearance, and improved pathological staging.

Multiple studies have underscored the oncological benefits of CME. Croner et al. [2] and Chow and Kim [3] highlighted that CME provides a standardized dissection technique that facilitates en bloc removal of lymphovascular structures, potentially reducing local recurrence. Dimitriou and Griniatsos [4] emphasized that CME yields a longer specimen, a larger mesocolic surface area, and a higher number of harvested lymph nodes compared to conventional surgery. Koh and Tan [5] noted that these features contribute to more accurate TNM staging and may influence long-term survival outcomes.

Despite these benefits, the technical demands of CME, particularly near the superior mesenteric vein (SMV) and its branches, have limited its widespread adoption. However, systematic reviews and population-based studies, including those by Bertelsen et al. [6], have validated its oncological safety and efficacy. Notably, the German S3 guidelines now recommend CME as a standard approach for right-sided colon cancers [7].

By contrast, conventional right hemicolectomy (CON) typically involves intermediate ligation of feeding vessels and dissection through a mesenteric window, without systematic attention to mesocolic integrity or CVL. As a result, it often yields fewer lymph nodes and may lack the oncological completeness offered by CME.

Given these evolving standards, this study was conducted to comparatively evaluate CME and CON in patients with right-sided colon cancer. Specific parameters assessed include lymph node harvest, pathological staging, operative time, type of anastomosis (intracorporeal vs. extracorporeal), stapling technique (Barcelona vs. Standard), and postoperative complications. The study was conducted in a high-volume tertiary care center over a four-year period, aiming to assess whether the adoption of CME offers measurable clinical and oncological advantages over the conventional approach in routine surgical practice.

## Materials and methods

This retrospective observational study was conducted at Sree Balaji Medical College & Hospital, a tertiary care center in Chennai, India, over a period of four years, from January 2021 to December 2024. All adult patients (>18 years) who underwent surgery for right-sided colon cancer, involving the cecum, ascending colon, or hepatic flexure, were eligible for inclusion. Patients presenting with distant metastasis, adjacent organ invasion, bowel obstruction, or bowel perforation were excluded to maintain uniformity in the study cohort.

Preoperative evaluation included colonoscopy with biopsy to confirm histological diagnosis, alongside complete blood counts, liver and renal function tests, and imaging studies. Contrast-enhanced computed tomography (CT) scans of the abdomen and pelvis were performed in all cases to assess the extent of the primary tumor and nodal involvement, and a CT chest was obtained to rule out pulmonary metastasis. All patients were discussed in a multidisciplinary tumor board prior to surgery. The baseline demographic characteristics of both groups are presented in Table 1. Data supported by standard colorectal cancer guidelines and staging protocols as referenced in Liang et al. [8] and Enker et al. [9].

**Table 1:** Preoperative assessment modalities Data supported by standard colorectal cancer guidelines and staging protocols as referenced in Liang et al. [8] and Enker et al. [9].

Investigations	Purpose
Colonoscopy with biopsy	Histological confirmation of malignancy
Complete blood count (CBC)	Evaluation of anemia and fitness for surgery
Liver and renal function tests (LFT, RFT)	Assessment of organ function
Contrast-enhanced CT abdomen and pelvis	Staging of the primary tumor and detection of lymph node involvement
CT chest	Evaluation for pulmonary metastases

Patients were categorized into two groups based on the surgical approach: CON (Group A) and CME (Group B). CON involved dissection through the mesentery with intermediate ligation of the ileocolic vessels without systematic central vascular ligation. Tumors at the hepatic flexure or proximal transverse colon underwent extended right hemicolectomy to achieve adequate oncological margins. By contrast, CME was performed using sharp dissection along the embryological mesocolic planes with central ligation of the ileocolic, right colic, and middle colic vessels at their origins. During CME, careful dissection around the superior mesenteric vein (SMV) and identification of Henle’s trunk were emphasized. Both open and laparoscopic approaches were utilized in both groups, with the choice depending on surgeon preference and patient characteristics. Table 2 compares postoperative complication rates between CME and conventional groups. Technical distinctions are based on the surgical descriptions detailed by Enker et al. [9], Tümay et al. [6], and Toyota et al. [10].

**Table 2:** Comparison of surgical techniques Technical distinctions are based on the surgical descriptions detailed by Enker et al. [9], Tümay et al. [6], and Toyota et al. [10].

Parameter	Conventional hemicolectomy	Complete mesocolic excision (CME)
Dissection plane	Non-anatomical mesenteric window	Sharp dissection along embryological planes
Vascular llgation	Intermediate	Central vascular ligation at vessel origins
Handling of Henle’s trunk	Not routinely identified	Carefully dissected and divided when necessary
Surgical approach	Open or laparoscopic	Open or laparoscopic
Margin distance maintained	≥10 cm	≥10 cm

**Figure 1:**
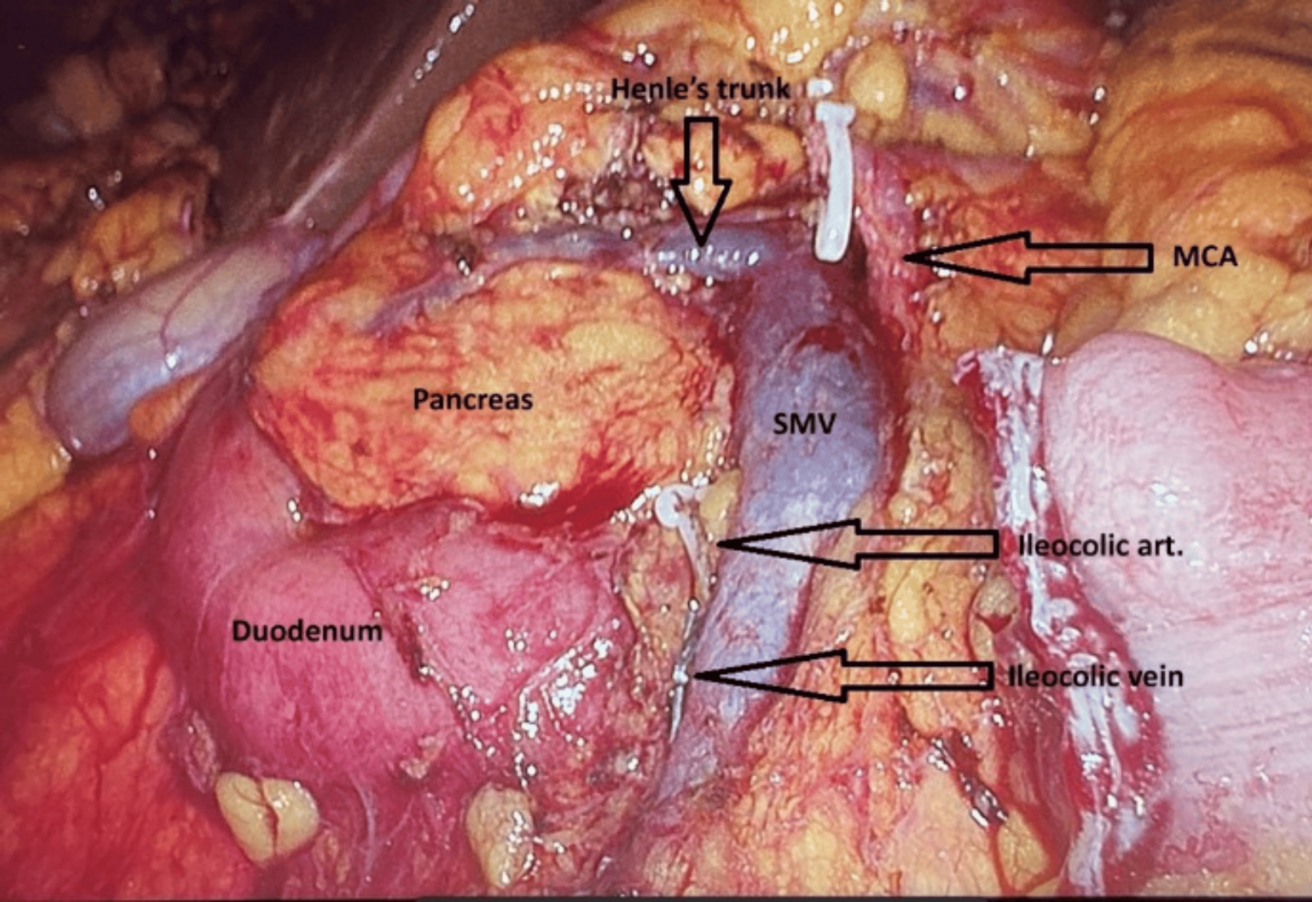
Intraoperative picture post complete mesocolic excision

This image (Figure 1) demonstrates a critical step in the execution of CME with central vascular ligation for right colon cancer. Key anatomical structures are clearly visualized: the superior mesenteric vein (SMV) is centrally exposed with identification of Henle's trunk, an important venous confluence draining the right gastroepiploic and superior right colic veins. The middle colic artery (MCA) and the Ileocolic artery and vein are also visualized at their origins, dissected off the SMV. The pancreas and duodenum form the posterior boundary of the dissection. This meticulous dissection highlights the central vascular ligation principle of CME, aimed at maximizing oncological clearance through en bloc resection of the mesocolon within intact embryological planes.

Anastomotic techniques included intracorporeal anastomosis (ICA) and extracorporeal anastomosis (ECA). ICA was more frequently performed in the CME group, utilizing minimally invasive techniques. Furthermore, two stapling techniques were utilized: the standard technique and the modified Barcelona technique, the latter involving a double-staple configuration that promotes a wide, isoperistaltic lumen. Operative outcomes such as blood loss and operative time are summarized in Table 3. Descriptions are adapted from Emile et al. [11], Brown et al. [12], and Sztipits et al. [13], which outline ICA, ECA, and the Barcelona technique.

**Table 3:** Types of anastomosis and stapling techniques Descriptions are adapted from Emile et al. [11], Brown et al. [12], and Sztipits et al. [13], which outline ICA, ECA, and the Barcelona technique.

Technique	Description
Intracorporeal anastomosis (ICA)	Anastomosis created within the abdominal cavity using laparoscopic instruments
Extracorporeal anastomosis (ECA)	Anastomosis created outside the body through a small abdominal incision
Standard stapling technique	Routine linear and circular stapler use for anastomosis
Barcelona technique	Modified double-staple technique favoring wide, isoperistaltic lumens

**Figure 2:**
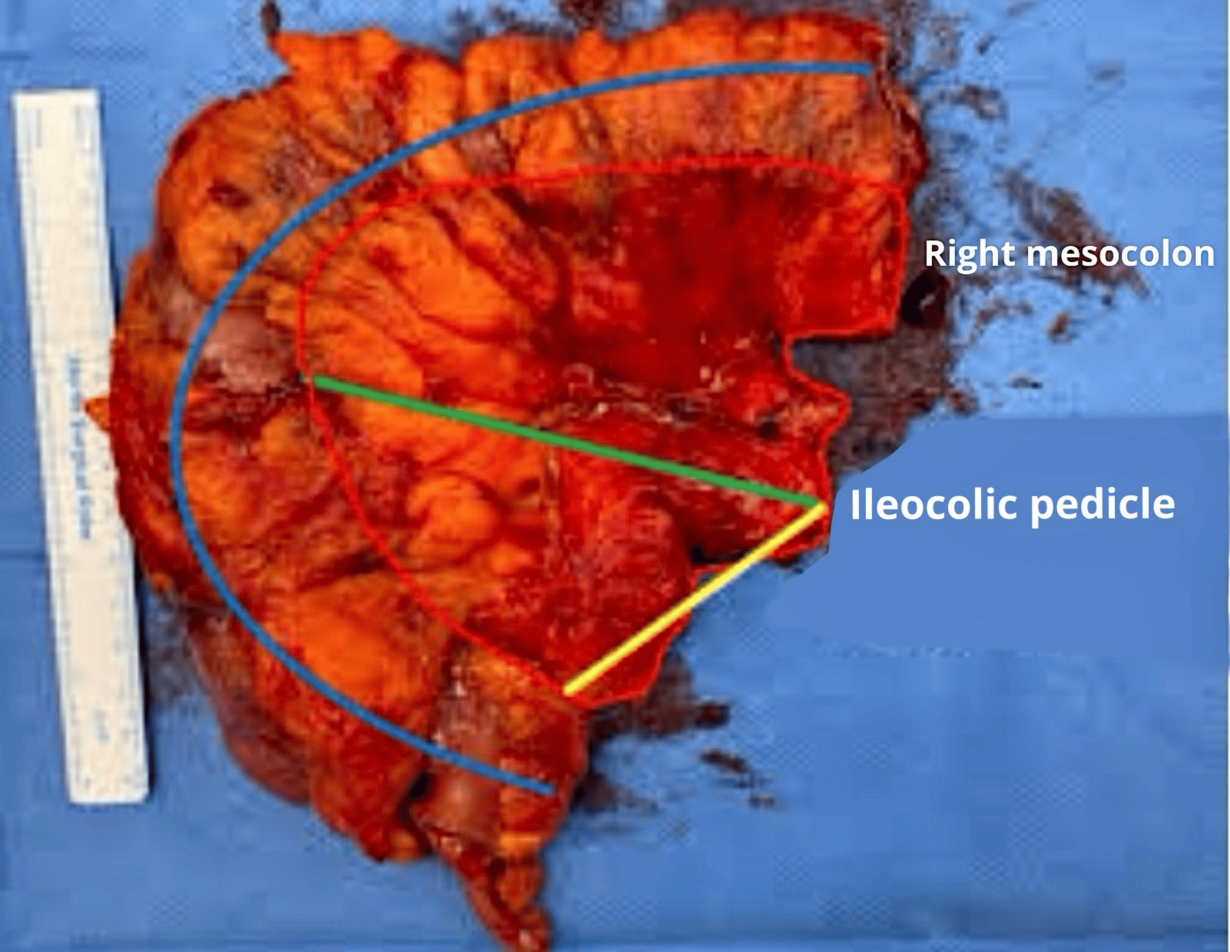
Resected right colon specimen demonstrating central vascular ligation and mesocolic integrity

**Figure 3:**
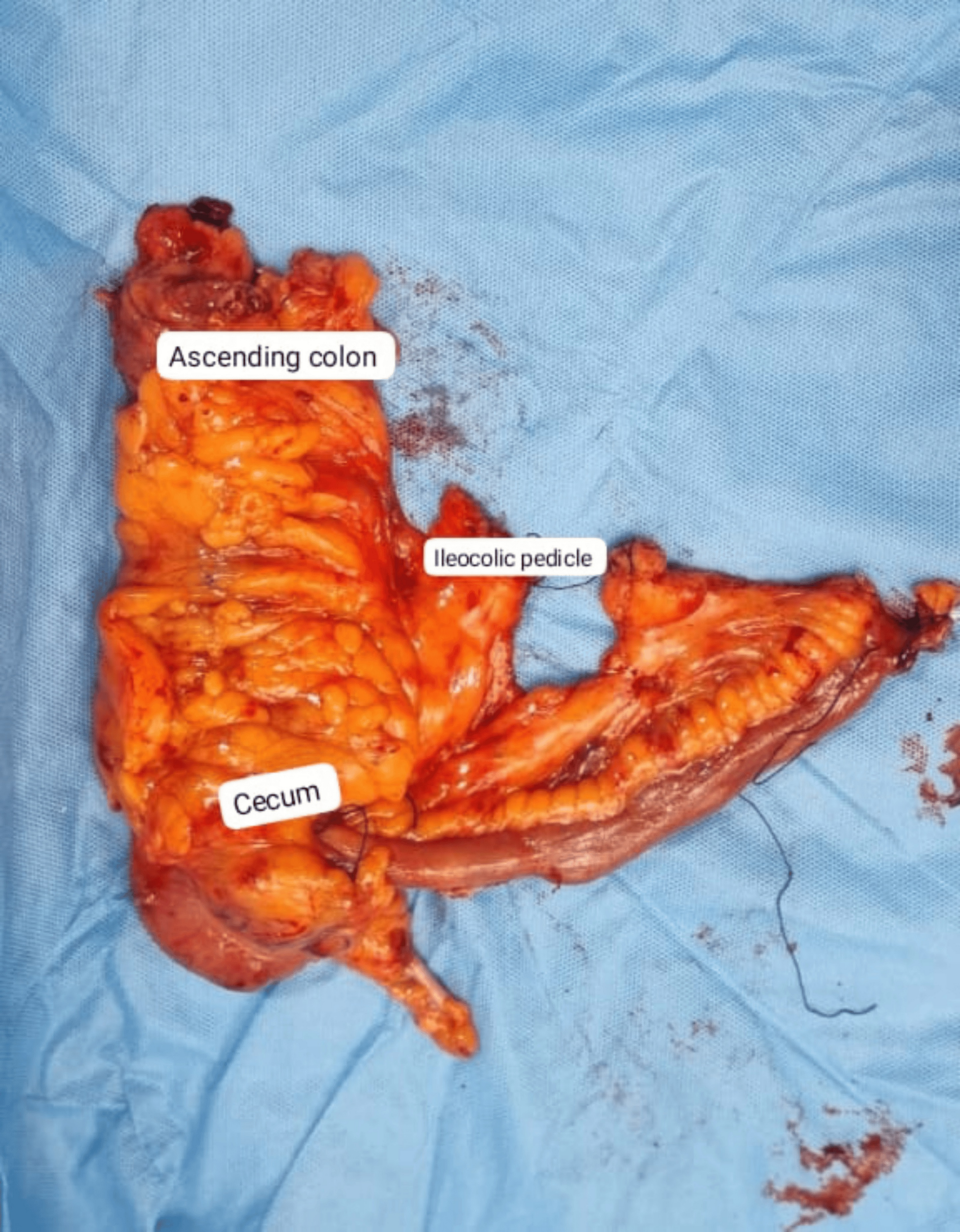
Right hemicolectomy specimen showing cecum, ascending colon, and ileocolic pedicle

**Figure 4:**
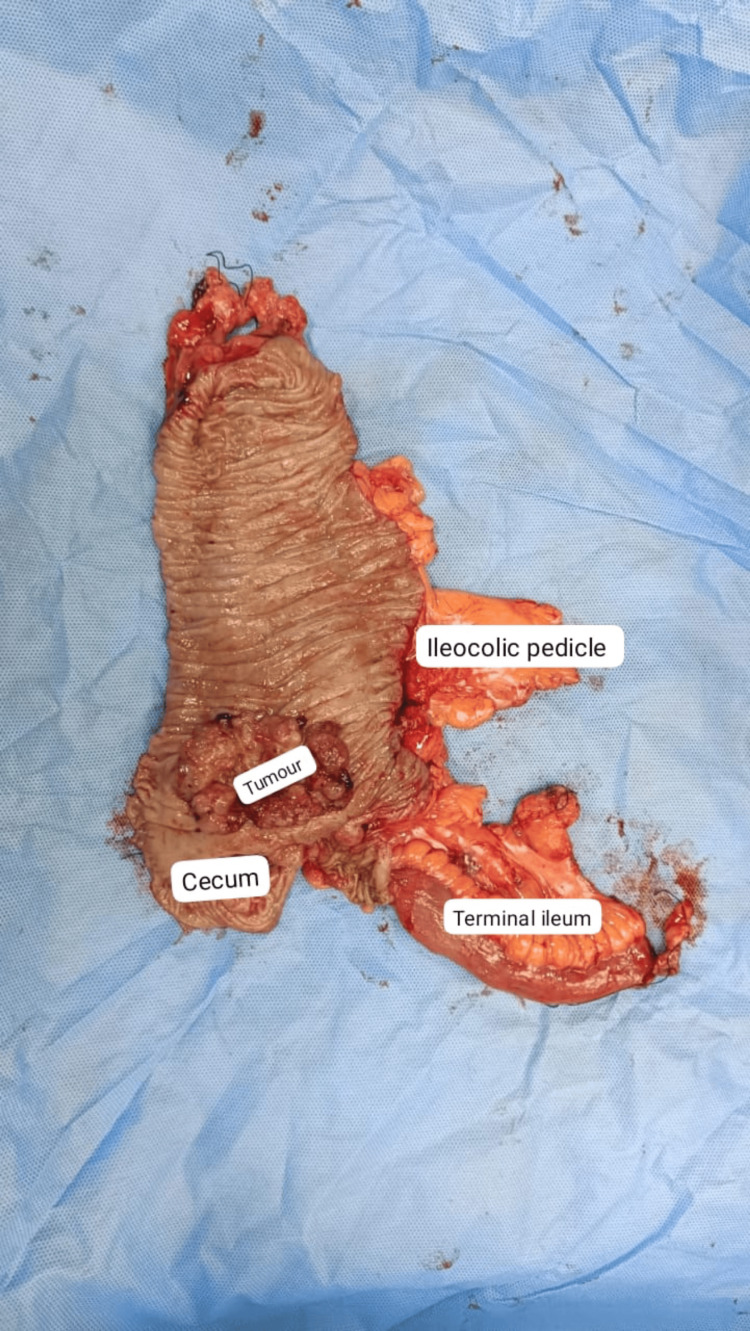
Gross specimen of right hemicolectomy showing tumor involving the cecum, terminal ileum, and ileocolic pedicle

All resected specimens were submitted for histopathological examination (Figures 2-4). Pathology assessment included tumor type, grade of differentiation, margin status, number of lymph nodes harvested, and the presence of lymph node metastasis. Postoperative outcomes were monitored, including surgical site infections, bleeding, anastomotic leaks, pulmonary infections, and paralytic ileus, both during hospital stay and outpatient follow-up visits.

Statistical analysis was performed using IBM SPSS Statistics for Windows, Version 26.0 (released 2019, IBM Corp., Armonk, NY). Categorical variables were compared using the Chi-square test or Fisher’s exact test, and continuous variables were compared using the independent t-test or Mann-Whitney U test, depending on data distribution. A p-value of less than 0.05 was considered statistically significant.

## Results

The study included 180 patients, with 86 undergoing CON and 94 undergoing CME. There were no significant differences in baseline demographics, including age, gender, and BMI, between the two groups (p-values of 0.374, 0.857, and 0.353, respectively). The distribution of tumor locations was also similar across both groups, with no significant differences observed (p = 0.442). When comparing surgical outcomes, the CME group showed a significantly higher lymph node yield, with 94.7% of patients having ≥12 lymph nodes harvested, compared to 79.1% in the CON group (p < 0.001). Although the operative time was longer in the CME group (156 ± 47 minutes vs. 112 ± 35 minutes), the difference was not statistically significant (p = 0.062). In addition, the CME group had a significantly higher adoption of intracorporeal anastomosis (44.7% vs. 14.0%, p = 0.001) and the Barcelona stapling technique (35.1% vs. 4.7%, p < 0.001). Postoperative complications, including bleeding, surgical site infections (SSI), and anastomotic leaks, were similar between the groups, with no significant differences in complication rates (p-values for all > 0.05). These findings suggest that while CME requires more time, it results in better lymph node harvest and a higher rate of minimally invasive techniques without increasing postoperative complications.

Table 4 compares various clinical parameters between the CON group (n = 86) and CME group (n = 94). Age, gender distribution, BMI, hemoglobin levels, and serum albumin levels were assessed. The results showed no statistically significant differences between the two groups for any of these parameters. Specifically, the mean age was slightly higher in the CON group (65.4 ± 12.5 years) compared to the CME group (62.6 ± 15.6 years), but the p-value of 0.374 (t = 0.89) indicates that this difference is not statistically significant. Similarly, gender distribution, with 58.1% males in the CON group and 61.7% in the CME group, was comparable between the groups, with a p-value of 0.857 (χ² = 0.03). BMI also showed no significant difference, with a mean of 27.3 ± 5.4 in the CON group and 26.3 ± 4.2 in the CME group (p-value = 0.353, t = 0.93). Hemoglobin levels were nearly identical (11.2 ± 1.5 g/dL vs. 11.4 ± 1.3 g/dL), with a p-value of 0.276 (t = -1.09). Lastly, serum albumin levels were also similar between the groups, with the CON group having 3.6 ± 0.4 g/dL and the CME group having 3.7 ± 0.5 g/dL, yielding a p-value of 0.321 (t = -1.00). Overall, these findings suggest that the two groups are comparable in terms of these baseline clinical parameters.

**Table 4:** Baseline demographics

Parameter	CON group (n = 86)	CME group (n = 94)	p-value	Test statistic
Age (years)	65.4 ± 12.5	62.6 ± 15.6	0.374	t = 0.89
Male (%)	58.1%	61.7%	0.857	χ² = 0.03
Female (%)	41.9%	38.3%		
BMI (kg/m²)	27.3 ± 5.4	26.3 ± 4.2	0.353	t = 0.93
Hemoglobin (g/dL)	11.2 ± 1.5	11.4 ± 1.3	0.276	t = -1.09
Serum albumin	3.6 ± 0.4	3.7 ± 0.5	0.321	t = -1.00

Table 5 compares the distribution of disease locations between the CON group and CME group, specifically focusing on the cecum, ascending colon, and hepatic flexure. For the cecum, 40.7% of cases in the CON group and 31.9% in the CME group were affected, with a p-value of 0.442 (χ² = 1.63), indicating no significant difference between the two groups. In the ascending colon, 34.9% of the cases in the CON group and 40.4% in the CME group were observed, again with a p-value of 0.442 (χ² = 1.63), showing no statistical significance. Lastly, for the hepatic flexure, 24.4% in the CON group and 27.7% in the CME group were affected, with the same p-value of 0.442 (χ² = 1.63), suggesting no significant difference in disease distribution. Overall, the p-values for all locations indicate that the differences between the two groups in terms of disease location are not statistically significant.

**Table 5:** Comparison of the locations of tumors in the two groups.

Location	CON (%)	CME (%)	p-value	Test statistic
Cecum	40.7%	31.9%	0.442	χ² = 1.63
Ascending colon	34.9%	40.4%	0.442	χ² = 1.63
Hepatic flexure	24.4%	27.7%	0.442	χ² = 1.63

Table 6 presents a comparison of tumor characteristics between the CON group and CME group, focusing on histological type, pathological stage, and lymph node involvement. For the histological type, the proportion of adenocarcinoma was 83.7% in the CON group (n = 72) and 85.1% in the CME group (n = 80), with a p-value of 0.538 (χ² = 0.38), indicating no significant difference between the groups. Similarly, mucinous carcinoma was found in 11.6% of the CON group (n = 10) and 10.6% of the CME group (n = 10), with the same p-value of 0.538 (χ² = 0.38), suggesting no significant difference. The incidence of signet ring cell carcinoma was 4.7% in the CON group (n = 4) and 4.3% in the CME group (n = 4), again showing no statistically significant difference (p-value = 0.538, χ² = 0.38). In terms of pathological staging, the proportions of patients in each stage were similar between the two groups. Pathological stage I was observed in 23.3% of the CON group (n = 20) and 23.4% of the CME group (n = 22), with a p-value of 0.481 (χ² = 1.46), indicating no significant difference. Pathological stage II was seen in 44.2% of the CON group (n = 38) and 44.7% of the CME group (n = 42), while 32.6% of the CON group (n = 28) and 31.9% of the CME group (n = 30) had pathological stage III. These differences were not statistically significant. A significant difference was observed in the number of lymph nodes examined, with 79.1% of the CON group (n = 68) and 94.7% of the CME group (n = 89) having ≥12 lymph nodes sampled (p-value < 0.001, χ² = 8.86), indicating a statistically significant higher proportion of patients in the CME group undergoing a more thorough lymph node examination. However, the lymph node positivity rate was similar between the groups, with 44.1% in the CON group (n = 38) and 42.5% in the CME group (n = 40), yielding a p-value of 0.891 (χ² = 0.02), indicating no significant difference in lymph node positivity. Overall, the results suggest that, except for the number of lymph nodes examined, there were no significant differences between the groups in terms of histological type, pathological stage, or lymph node positivity.

**Table 6:** Histopathology and nodal status

Parameter	CON group	CME group	p-value	Test statistic
Adenocarcinoma	83.7% (n = 72)	85.1% (n = 80)	0.538	χ² = 0.38
Mucinous carcinoma	11.6% (n = 10)	10.6% (n = 10)	0.538	χ² = 0.38
Signet ring cell	4.7% (n = 4)	4.3% (n = 4)	0.538	χ² = 0.38
Pathological stage I	23.3% (n = 20)	23.4% (n = 22)	0.481	χ² = 1.46
Pathological stage II	44.2% (n = 38)	44.7% (n = 42)		
Pathological stage III	32.6% (n = 28)	31.9% (n = 30)		
Lymph nodes ≥12	79.1% (n = 68)	94.7% (n = 89)	<0.001	χ² = 8.86
Lymph node positivity	44.1% (n = 38)	42.5% (n = 40)	0.891	χ² = 0.02

Table 7 compares the surgical parameters between the CON group and CME group, focusing on surgery duration, anastomosis type, and stapling techniques. The duration of surgery was slightly shorter in the CON group (112 ± 35 minutes) compared to the CME group (156 ± 47 minutes), but the difference was not statistically significant (p-value = 0.062, t = -1.88). In terms of anastomosis type, intracorporeal anastomosis (ICA) was significantly more common in the CME group (44.7%, n = 42) compared to the CON group (14.0%, n = 12) (p-value = 0.001, χ² = 11.16). Conversely, extracorporeal anastomosis (ECA) was more frequent in the CON group (86.0%, n = 74) than in the CME group (55.3%, n = 52). Regarding stapling techniques, the Barcelona stapling technique was used in 35.1% of the CME group (n = 33) but only 4.7% of the CON group (n = 4), with a highly significant difference (p-value < 0.001, χ² = 27.38). The standard stapling technique was more commonly used in the CON group (95.3%, n = 82) compared to the CME group (64.9%, n = 61). In conclusion, there were significant differences in the type of anastomosis and stapling technique between the two groups, but the duration of surgery did not differ significantly.

**Table 7:** Surgical outcomes

Parameter	CON group	CME group	p-value	Test statistic
Duration of surgery (min)	112 ± 35	156 ± 47	0.062	t = -1.88
Intracorporeal anastomosis (ICA)	14.0% (n = 12)	44.7% (n = 42)	0.001	χ² = 11.16
Extracorporeal anastomosis (ECA)	86.0% (n = 74)	55.3% (n = 52)		
Barcelona stapling technique	4.7% (n = 4)	35.1% (n = 33)	<0.001	χ² = 27.38
Standard stapling technique	95.3% (n = 82)	64.9% (n = 61)		

Table 8 compares the postoperative complications between the conventional (CON) group (n = 86) and the CME group (n = 94). For bleeding, 4.7% in the CON group and 3.2% in the CME group experienced bleeding (p-value = 0.544, χ² = 0.37), with no significant difference. Wound site infection occurred in 7.0% of the CON group and 5.3% of the CME group (p-value = 0.622, χ² = 0.24), also showing no significant difference. Anastomotic leak was seen in 2.3% of the CON group and 1.1% of the CME group (p-value = 0.412, χ² = 0.67), indicating no significant difference. Pulmonary infection was noted in 3.5% of the CON group and 2.1% of the CME group (p-value = 0.392, χ² = 0.73), with no significant difference. Lastly, paralytic ileus occurred in 4.7% of the CON group and 2.1% of the CME group (p-value = 0.337, χ² = 0.92), showing no significant difference. In conclusion, the complication rates were similar between the two groups, with no statistically significant differences in any of the complications.

**Table 8:** Postoperative complications

Complication	Conventional (n = 86)	CME (n = 94)	p-value	Test statistic
Bleeding	4 (4.7%)	3 (3.2%)	0.544	χ² = 0.37
Wound site infection	6 (7.0%)	5 (5.3%)	0.622	χ² = 0.24
Anastomotic leak	2 (2.3%)	1 (1.1%)	0.412	χ² = 0.67
Pulmonary infection	3 (3.5%)	2 (2.1%)	0.392	χ² = 0.73
Paralytic Ileus	4 (4.7%)	2 (2.1%)	0.337	χ² = 0.92

## Discussion

This study presents a comparative evaluation of CME and CON in the management of right-sided colon cancer, analyzing data from 180 patients. It offers valuable insights into short-term surgical, pathological, and early postoperative outcomes, with particular focus on lymph node yield, positivity, complication rates, and operative details. Baseline characteristics such as age, sex, BMI, and nutritional parameters (hemoglobin and albumin) were comparable between groups, with no statistically significant differences. This ensures that the patient profiles were well matched, minimizing demographic bias and allowing for meaningful comparison.

Tumor location (cecum, ascending colon, and hepatic flexure) and histologic subtype (adenocarcinoma, mucinous carcinoma, and signet ring cell) were similarly distributed, and pathological staging showed no significant differences between groups. These similarities affirm that differences in surgical or oncologic outcomes can be attributed to the technique rather than underlying tumor biology or location. A major finding of this study is the significantly higher lymph node yield in the CME group compared to the CON group (p < 0.001), reaffirming the oncologic effectiveness of CME in achieving a radical mesocolic dissection. This is consistent with findings from Tümay et al. [6], who reported a median lymph node yield of 58 in CME versus 31 in CON (p < 0.001), and Sztipits et al. [13], who found significantly more patients achieving ≥19 lymph nodes in CME (74%) compared to CON (53%) (p = 0.041). In terms of anastomotic technique, our study observed a significantly greater use of intracorporeal anastomosis (ICA) in the CME group (44.7%) compared to the CON group (14.0%), which aligns with literature reporting the benefits of ICA in reducing bowel traction, improving visualization, and facilitating quicker recovery [8,9].

In addition, the Barcelona stapling technique was employed more frequently in the CME group (35.1% vs 4.7%, p < 0.001). This technique promotes isoperistaltic, wide-lumen anastomosis with minimal angulation and has been associated with a lower risk of postoperative ileus and potentially fewer anastomotic complications, although not statistically significant in our study [9]. While the lymph node positivity rate remained similar (CME: 42.5%, CON: 44.1%, p = 0.891), the higher lymph node harvest in CME enhances staging accuracy. This is supported by the RELARC [9] and COLD [10] trials, which also report improved nodal yield and safety in CME procedures without increased morbidity. Foundational studies by Enker et al. [9] first demonstrated the survival benefit of wide anatomic resection in colorectal cancer. This concept was further reinforced by Le Voyer et al. [14], who linked higher node counts to improved survival, and Johnson et al. [15], who emphasized the prognostic value of negative lymph nodes. Liang et al. [8] echoed similar benefits of extended lymphadenectomy in their study on laparoscopic D3 dissection. Furthermore, Toyota et al. [10] underscored the anatomical rationale and oncologic importance of extended lymph node dissection in right colon cancer, supporting the surgical foundation of CME [15].

One of the key limitations of our study is the limited control over population sampling inherent in retrospective study designs. In our cohort, patients undergoing conventional right hemicolectomy were predominantly older, with multiple comorbidities and higher ASA scores (ASA-3), while those selected for CME were typically younger, had fewer comorbid conditions, and lower ASA scores (ASA-1 or 2). This imbalance introduces a potential selection bias, which may influence the observed outcomes and limit the comparability between the groups. Future prospective, randomized studies would help mitigate these biases and provide a more robust assessment of outcomes across surgical techniques.

In conclusion, this study supports CME as a technically sound and oncologically superior approach for right-sided colon cancer. Its integration with intracorporeal anastomosis and the Barcelona stapling technique further improves surgical outcomes, particularly in centers equipped with advanced laparoscopic or robotic capabilities.

## Conclusions

CME with central vascular ligation demonstrated superior lymph node yield and increased adoption of minimally invasive and advanced anastomotic techniques compared to CON. The CME group showed significantly greater use of intracorporeal anastomosis and the Barcelona stapling technique, both of which are associated with enhanced anatomical precision, improved visualization, and potentially better functional outcomes in selected patients. Despite a longer operative duration, CME did not result in increased short-term morbidity, supporting its feasibility and safety in high-volume surgical centers. When performed by trained teams, CME offers oncological completeness, accurate staging, and better standardization of colorectal cancer surgery. Thus, CME can be considered a surgically and oncologically favorable alternative to conventional hemicolectomy, especially when combined with minimally invasive approaches and refined anastomotic techniques.
